# The cohesin-like RecN protein stimulates RecA-mediated recombinational repair of DNA double-strand breaks

**DOI:** 10.1038/ncomms15282

**Published:** 2017-05-17

**Authors:** Lee A. Uranga, Emigdio D. Reyes, Praveen L. Patidar, Lindsay N. Redman, Shelley L. Lusetti

**Affiliations:** 1 Department of Chemistry and Biochemistry, New Mexico State University, P.O. Box 30001, MSC 3C, Las Cruces, New Mexico 88003, USA

## Abstract

RecN is a cohesin-like protein involved in DNA double-strand break repair in bacteria. The RecA recombinase functions to mediate repair via homologous DNA strand invasion to form D-loops. Here we provide evidence that the RecN protein stimulates the DNA strand invasion step of RecA-mediated recombinational DNA repair. The intermolecular DNA tethering activity of RecN protein described previously cannot fully explain this novel activity since stimulation of RecA function is species-specific and requires RecN ATP hydrolysis. Further, DNA-bound RecA protein increases the rate of ATP hydrolysis catalysed by RecN during the DNA pairing reaction. DNA-dependent RecN ATPase kinetics are affected by RecA protein in a manner suggesting a specific order of protein–DNA assembly, with RecN acting after RecA binds DNA. We present a model for RecN function that includes presynaptic stimulation of the bacterial repair pathway perhaps by contributing to the RecA homology search before ternary complex formation.

## Abstract (web summary)

RecN is a cohesin-like molecule involved in the bacterial response to DNA double-strand breaks. Here the authors provide evidence that RecN stimulates the DNA strand invasion step of RecA-mediated recombination.

DNA double-strand break (DSB) repair is critical for chromosomal maintenance. There are several proposed pathways for homology-directed DSB repair including single-strand annealing, synthesis-dependent strand annealing, break-induced replication and Holliday junction pathways^1^. All recombinational DSB repair pathways have at least one step in common: the pairing of a broken DNA end with an intact part of a homologous chromosome. This central DNA strand invasion step is carried out by a family of enzymes that include the bacterial RecA and the eukaryotic Rad51 proteins^2^,^3^. While many of the functions of gene products involved in various DSB pathways are known, there are still key players, even in bacteria, which remain enigmatic. One such mysterious protein critical to DSB repair in bacteria is the RecN protein.

The RecN protein is a member of the structural maintenance of chromosomes (SMC) family of proteins. SMC proteins have important functions in a variety of housekeeping DNA processes including chromosomal condensation, sister chromatid cohesion and recombinational DNA repair^4^. The *Deinococcus radiodurans* RecN protein was shown previously to act as a cohesin mediating the intermolecular tethering of DNA molecules^5^, confirming an SMC protein-like function for a bacterial recombination enzyme. The SMC-like architecture of *D. radiodurans* RecN has further been confirmed by X-ray structure analysis^6^.

*Escherichia coli* RecN is involved in the recombinational repair of DNA damage and is likely functioning in both the RecFOR- and RecBCD-dependent pathways^7^. RecN forms discrete foci in response to DSBs in bacteria^8^,^9^,^10^ and the repair of DBSs is severely hampered in the absence of RecN^11^,^12^,^13^. But, what is the function of the RecN protein in recombinational DNA repair pathways? *Bacillus subtilis* RecN protein is thought to coordinate damaged DNA and recombination proteins early in the DNA damage response^14^,^15^ and RecN appears important for ultraviolet-induced compaction of the *E. coli* nucleoid^16^. Further, strains harbouring *recN* mutants are more sensitive to ionizing radiation or multiple, site-specific DSBs than to a single site-specific DSB in *E. coli.*^11^ These observations are consistent with a pre-recombination, chromosomal maintenance role for RecN protein.

The DNA tethering activity of RecN likely provides an important utility leading to DNA repair. However, evidence also suggests that RecN acts with or affects the RecA protein. First, *E. coli recN recA* double mutants are no more sensitive to DSBs than *recA* single mutants^11^, consistent with RecN involvement in RecA-mediated recombinational DNA repair. And further, it appears that RecA recruits RecN to DSBs in *E. coli*^9^ suggesting a role for RecN after RecA accumulates at a the site of damage. There are no published accounts that the *E. coli* RecN protein has been purified for biochemical studies, although not for a lack of trying. *E. coli* RecN is highly susceptible to proteolytic breakdown and is generally insoluble in aqueous solutions^17^,^18^. In the current study, we demonstrate the effect of the RecN protein on RecA-mediated recombination using a biochemical system and the purified *D. radiodurans* proteins. Here we report a significant stimulation of the RecA-mediated DNA strand invasion step of recombination by the RecN protein. Further, we establish the order of assembly of RecN and RecA proteins that leads to a functional interaction between these proteins. And, finally, we describe the influence of RecA on the ATP hydrolysis activity of the RecN protein.

## Results

### RecN protein stimulates RecA-mediated DNA strand exchange

**Figure 1:**
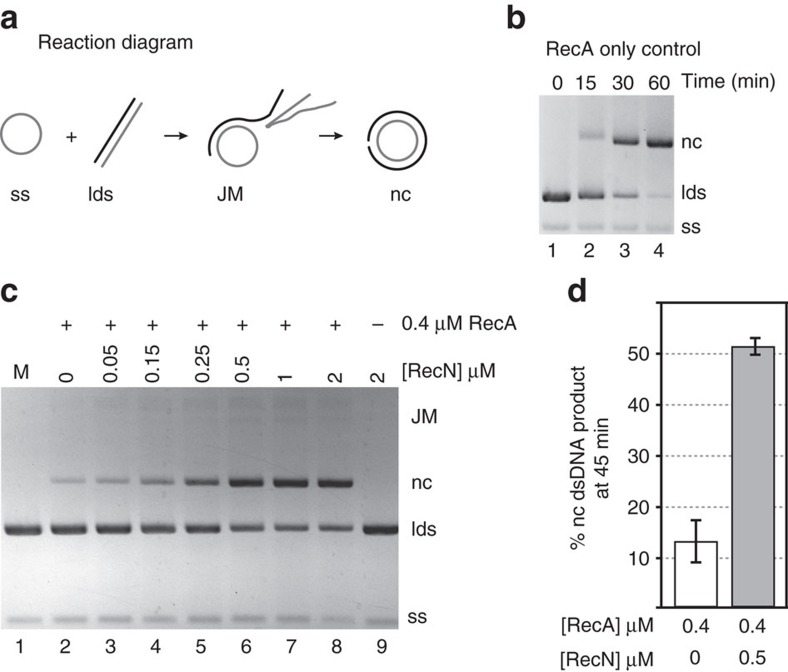
RecN stimulates RecA-mediated DNA three-strand exchange reactions. (**a**) Schematic of RecA-mediated DNA strand exchange reaction. RecA filaments formed on circular ssDNA (ss) invade and search for homology within linear duplex DNA (lds). The homology between the ssDNA bound by RecA and the duplex DNA is aligned. RecA exchanges these homologous strands forming intermediate, joint heteroduplex DNA molecules (JM). The intermediate joint molecule contains a three-stranded branch point that migrates the length of the molecule until nicked, circular duplex products (nc) are formed. The abbreviations described here (ss, lds, JM and nc) reflect the agarose gel labels used here and in subsequent figures. (**b**) RecA only control (normal conditions). RecA protein (5 μM) was incubated with 15 μM circular ssDNA for 10 min. ATP (3 mM) and SSB (1.5 μM) were added and incubated for an additional 10 min. Reactions were initiated by addition of homologous ldsDNA (15 μM) and incubated for the time indicated. The total reaction volume was 20 μl. (**c**) Reactions were carried out as described for **b** except under dilute conditions. The final concentrations of RecA, SSB, ssDNA and ldsDNA were 0.4, 0.08, 1 and 2 μM, respectively, and the total reaction volume was 120 μl. RecN protein was added at the concentration noted in the figure with the ATP and SSB. All reactions were stopped 45 min after the addition of ldsDNA except for M (stopped immediately after ldsDNA addition). DNA was recovered from the reaction before gel electrophoresis (see Methods). This experiment was repeated three times with similar results. (**d**) Quantification of amount of nc duplex DNA product formed by 0.4 μM RecA protein in 45 min in the presence or absence of 0.5 μM RecN protein. The band intensity of the product was divided by the sum of the band intensities of all duplex DNA species in the same gel lane, as detected by the TotalLab gel quantification software. Error bars represent the s.d. of five independent experiments.

The RecN protein can actively reduce the in-solution distance between DNA molecules^5^,^19^,^20^. We reasoned that intermolecular DNA tethering might stimulate RecA-mediated DNA strand exchange under dilute, suboptimal reaction conditions. Therefore, the effect of *D. radiodurans* RecN protein on RecA activity was assayed *in vitro* using a modified DNA strand exchange reaction (Fig. 1a). The reaction is carried out in the order typically used to assess RecA-promoted DNA strand exchange. However, under the dilute DNA conditions (see Methods) used for reactions shown in Fig. 1c, RecA protein only promotes a small amount of nicked, circular duplex product compared to the reaction carried out under standard (undiluted) DNA conditions (compare lane 2 of **c** with lane 4 of **b** in Fig. 1). We found that the addition of RecN protein to this reaction greatly stimulates product formation in a concentration-dependent manner (Fig. 1c). The stimulation is observed with as little as 250 nM RecN protein and saturates with the addition of 1 μM RecN protein. The addition of 500 nM RecN protein stimulates product formation by RecA protein more than threefold under these conditions (Fig. 1d). RecN protein alone does not promote DNA strand exchange (lane 9, Fig. 1c).

### RecN protein stimulates RecA-mediated D-loop formation

**Figure 2:**
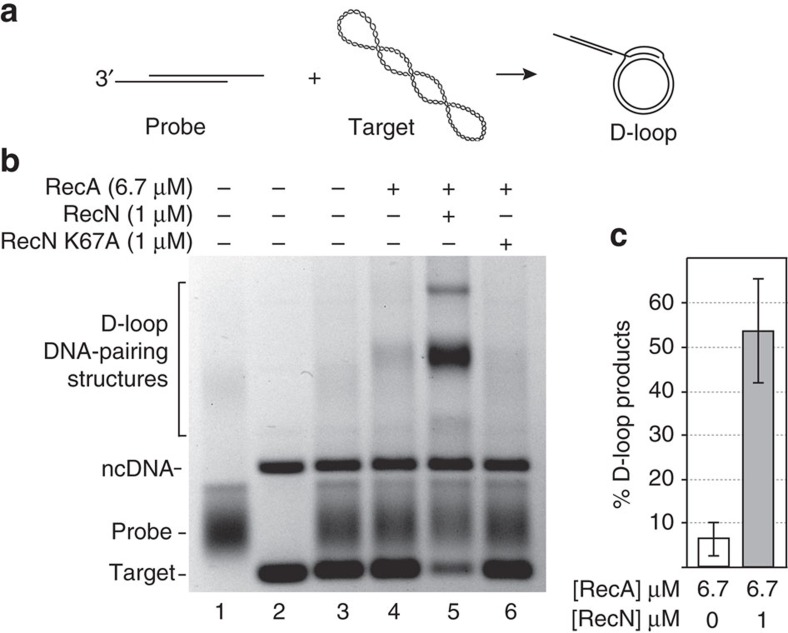
RecN stimulates RecA-dependent D-loop formation. (**a**) Schematic of RecA-dependent D-loop reaction. RecA filaments formed on the linear duplex plasmid DNA substrate containing 150-nucleotide (nt) 3′-ssDNA overhangs (probe) promote strand invasion within the 2.4 kb, homologous, supercoiled plasmid DNA (target). RecA exchanges the homologous strands forming D-loop structures. These descriptions, probe, target and D-loop, reflect the agarose gel labels used here and in subsequent figures. (**b**) RecA (6.7 μM) was incubated with 20 μM probe DNA for 10 min. ATP (3 mM) and 1 μM RecN or RecN K67A mutant, as indicated at the top of each lane, were incubated for an additional 10 min before starting the reaction with the addition of 20 μM homologous target DNA. All reactions were incubated for 45 min. (**c**) Quantification of amount of D-loop-pairing structures formed by 6.7 μM RecA protein in 45 min in the presence or absence of 1 μM RecN protein. The D-loop products are defined as the sum of all DNA band intensities in a particular lane that correspond to the mobility of the D-loop DNA-pairing structures identified in **b** that were detected by the TotalLab gel quantification software. This sum was divided by the sum of all band intensities (except the band corresponding the ncDNA) in the same lane. Error bars represent the s.d. of six independent experiments.

**Figure 3:**
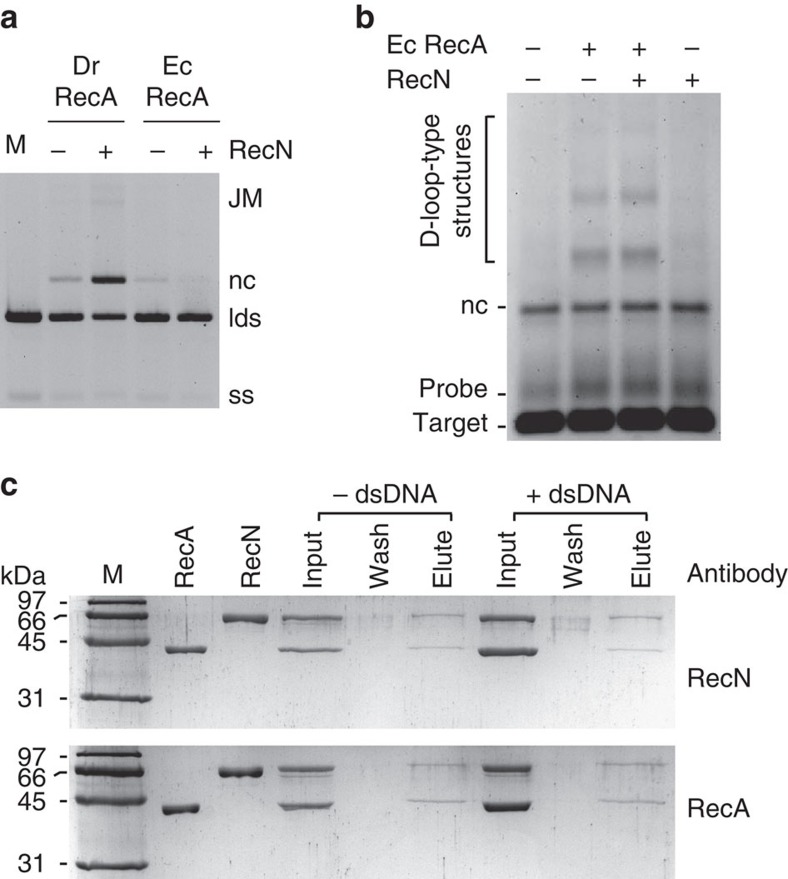
RecA and RecN proteins interact. (**a**) The *D. radiodurans* (Dr) or *E. coli* (Ec) RecA protein (0.4 μM) was incubated with 1 μM circular ssDNA (ss) for 10 min. ATP (3 mM), RecN (0.5 μM, where indicated) and 0.08 μM SSB were added and incubated for an additional 10 min. The reaction was initiated by the addition of 2 μM homologous duplex DNA (lds). All reactions were incubated for 45 min after lds addition except for M. The reaction of the control lane (M) was immediately stopped after lds addition. DNA was recovered from the reaction before gel electrophoresis (see Methods). This experiment was repeated three times with similar results. We observed no measurable difference in experiments with EcRecA+or −DrRecN protein. Quantification of RecN stimulation of DrRecA DNA stand exchange under these conditions is included in Fig. 2c. (**b**) EcRecA (6.7 μM) was incubated with 20 μM probe DNA for 10 min. ATP (3 mM) and 1 μM RecN, as indicated at the top of each lane, were added and incubated for an additional 10 min. The reactions were initiated by the addition of 20 μM target DNA. All reactions were incubated for 45 min. See Fig. 2 for target and probe DNA description. This experiment was repeated three times with no measurable difference between + and – RecN conditions. (**c**) Purified *D. radiodurans* RecA (38 kDA) and RecN (60 kDa) proteins co-elute, in the presence (+dsDNA) or absence (−dsDNA) of linear duplex DNA, from a RecN antibody-coupled resin (top) or from a RecA antibody-coupled resin (bottom). Lane M indicates a protein size marker. The input lanes contain an 8 μl load of a mixture of 0.12 μg RecN per μl and 0.08 μg RecA per μl. Excess protein complex was removed during the early wash steps, and 8 μl of the final 50 μl wash and 8 μl of the 50 μl elution were loaded directly onto the gel.

As described in the introduction, evidence suggests that RecN may function at an early step in the DSB repair pathway. Figure 1 shows that RecN indeed stimulates RecA-promoted DNA strand exchange *in vitro*. This enhancement is likely accomplished by RecN bringing the recombining substrates closer together enabling RecA to find and pair the homologous DNA molecules. To clearly show that RecN acts early in the reaction, we confirmed the stimulatory effect of adding RecN to a RecA-mediated displacement loop (D-loop) reaction (Fig. 2). D-loops form when RecA filament promotes synapsis of a 3′-overhang on the probing DNA sequence with a complementary region of a homologous target duplex DNA molecule (Fig. 2a). This type of reaction is thought to mimic the initial pairing step of recombinational DSB repair^3^. Products are observable independent of the extensive DNA branch migration required to detect the products of the DNA strand exchange reaction shown in Fig. 1. The inefficient D-loop formation mediated by the RecA protein alone (lane 4, Fig. 2b) is significantly enhanced by the addition of the RecN protein (lane 5, Fig. 2b,c). RecN does not promote DNA pairing in the absence of RecA (see Fig. 3b below). Interestingly, the RecN stimulation of RecA-mediated D-loop formation is not observed when a RecN ATPase-deficient mutant (RecN K67A) is used in the reaction (lane 6, Fig. 2b). This indicates that RecN ATP hydrolysis is required for the stimulation of RecA under these conditions. The requirement for ATP hydrolysis will be explored further below.

### DNA pairing stimulated by RecN protein is species-specific

The RecN-dependent increase in DNA-pairing products promoted by the RecA protein shown in Figs 1 and 2 can be explained by a simple model in which the cohesin-like activity of RecN^5^ reduces the distance between substrate DNA molecules. This model predicts that RecN protein should enhance the DNA-pairing activity of any RecA recombinase. We tested the DNA strand exchange activity of the *E. coli* RecA protein in the presence of RecN (Fig. 3). Surprisingly, we observed no stimulation of the *E. coli* RecA protein by the RecN protein using either the diluted DNA strand exchange reaction (Fig. 3a) or the D-loop assay (Fig. 3b). This negative result may reflect the minor mechanistic differences between the *E. coli* RecA and *D. radiodurans* RecA proteins previously described^21^,^22^. It may also reflect a species-specific interaction. We explored the intriguing possibility of a direct interaction between purified RecA and RecN proteins using a pull-down strategy (Fig. 3c). The RecA and RecN proteins (2 μM each) were incubated in the presence or absence of DNA. Diluted mixtures were loaded onto RecN or RecA antibody covalently coupled resin, and unbound proteins were removed. On elution, RecA and RecN co-eluted suggesting a physical interaction. The RecA and RecN proteins do not interact nonspecifically with RecN and RecA antibodies, respectively (Supplementary Fig. 1). A direct interaction may explain the fact that *D. radiodurans* RecN does not stimulate the DNA strand exchange reactions catalysed by *E. coli* RecA protein (Fig. 3a,b), but does stimulate its cognate RecA protein (Figs 1 and 2).

### RecA bound to DNA stimulates RecN ATP hydrolysis

**Figure 4:**
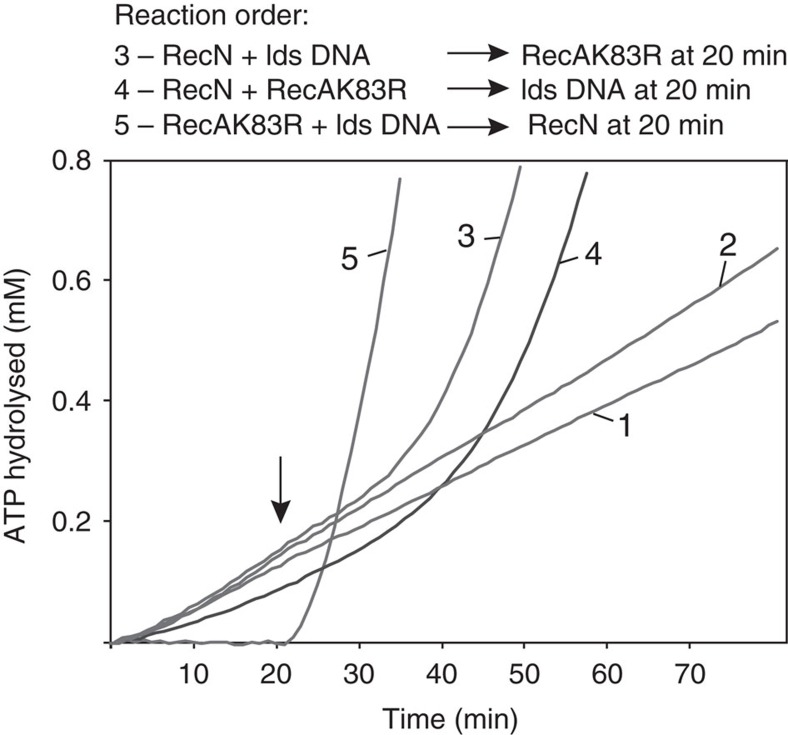
RecA protein stimulates the DNA-dependent rate of RecN ATP hydrolysis. ATPase reactions were carried out as described in the Methods section. The ATP hydrolysis measured reflects only that catalysed by RecN protein since RecA K83R is a ATPase-deficient RecA mutant. Linearized pEAW324 plasmid DNA is utilized where indicated. Reactions 1 and 2 are control experiments measuring the amount of ATP hydrolysis over time by RecN protein (2 μM) in the absence (reaction 1) or presence of 50 μM DNA (reaction 2). The order of addition for reactions 3, 4 and 5 are shown (top). The first set (reaction 3, RecN and DNA; reaction 4, RecN and RecA K83R; and reaction 5, RecA K83R and DNA) were incubated with 2.5 mM ATP in buffer N (see Methods) for 20 min before the second addition (reaction 3, RecA K83R; reaction 4, DNA; and reaction 5, RecN). The time of the second addition is indicated by a vertical arrow. The final concentration of RecN, RecA K83R and DNA was 2, 2 and 50 μM, respectively. See Table 1 for steady-state rates of RecN ATP hydrolysis and lag times.

The role of ATP hydrolysis by the RecN protein and cohesins in general is not well understood. Robert Lloyd and colleagues have shown that *recN* ATPase-deficient mutant bacterial strains (replacing lysine with alanine in the Walker A motif) have phenotypes that mimic that of a *recN* null mutant^23^. The purified RecN K67A mutant protein from *D. radiodurans* exhibits no measurable ATPase activity but is not defective in DNA binding or cohesin-like DNA-bridging activity (Supplementary Fig. 2). We were intrigued by the observation that RecN K67A mutant protein does not stimulate RecA-mediated DNA pairing (lane 6, Fig. 2b) especially given the fact ATP nucleotide is not required for the wild-type RecN protein to carry out cohesin-like DNA-bridging activity^5^. In an attempt to understand the role of ATP hydrolysis, we explored the effect of RecA protein on the ATP hydrolysis activity of RecN (Fig. 4). We showed previously that the rate of ATP hydrolysis catalysed by the RecN protein is not stimulated by single-stranded DNA (ssDNA), but is stimulated by linear, and relaxed or supercoiled circular duplex DNA^5^. The experiments shown in Fig. 4 are carried out at a sub-optimal concentration ratio of RecN to DNA such that the DNA-independent and -dependent ATP hydrolysis rates are similar (reactions 1 and 2 of Fig. 4, Table 1 and ref. 5). Since RecA also hydrolyses ATP, we utilized the ATPase-deficient *D. radiodurans* RecA Walker A mutant protein (RecA K83R) to test the effect of added RecA protein under the conditions previously optimized for RecN activity^5^. We observed a three- to fivefold increase in the RecN ATP hydrolysis rate when a ternary mixture of RecN (2 μM), RecA K83R (2 μM) and duplex DNA (50 μM) are present in the reaction mixture (compare reactions 3, 4 and 5 with reactions 1 and 2, Fig. 4 and Table 1). This indicates that RecN ATPase activity is enhanced in the presence of RecA protein. Data in Supplementary Fig. 3 confirm that the stimulation of RecN ATP hydrolysis is species-specific and occurs under conditions optimized for RecA protein activity.

The RecN ATPase rate increase is more instructive when we measure the time required for RecN protein to reach a steady-state rate of ATP hydrolysis (lag time) as a function of protein order of addition. When RecN is allowed to interact with the DNA for 20 min before the addition of RecA, a lag of 25 min is observed (reaction 3, Fig. 4 and Table 1). The lag increases to more than 30 min when RecN and RecA are pre-incubated before the addition of duplex DNA (reaction 4, Fig. 4). In this case, there is a small but reproducible decrease in the DNA-independent ATPase rate of RecN before the ternary mixture is complete by the addition of DNA (compare reactions 1 and 4, Fig. 4). This may be due to the interactions between RecA and RecN proteins. Finally, the lag in RecN ATP hydrolysis described above is almost eliminated if RecN protein is added to DNA pre-incubated with RecA protein (reaction 5, Fig. 4). The final, steady-state rate of RecN-catalysed ATP hydrolysis is similar in all three orders of addition (Table 1).

The most likely explanation for the lag measured when either RecN is pre-incubated with DNA before the addition of RecA (reaction 3, Fig. 4) or RecN is pre-incubated with RecA before the addition of DNA (reaction 4, Fig. 4) is that a reorganization of proteins is occurring such that RecN is dissociating from DNA or RecA so that RecA is free to bind the DNA. And, the increase in RecN activity only occurs when RecN interacts with DNA that is bound with RecA. Consequently, we observe no lag to RecN ATP hydrolysis when RecN is added to preformed RecA filaments. These data suggest that RecN ATPase activity may be needed for a step in the DSB repair pathway after RecA is loaded onto the DNA.

### RecN ATPase is stimulated under RecA D-loop conditions

**Figure 5:**
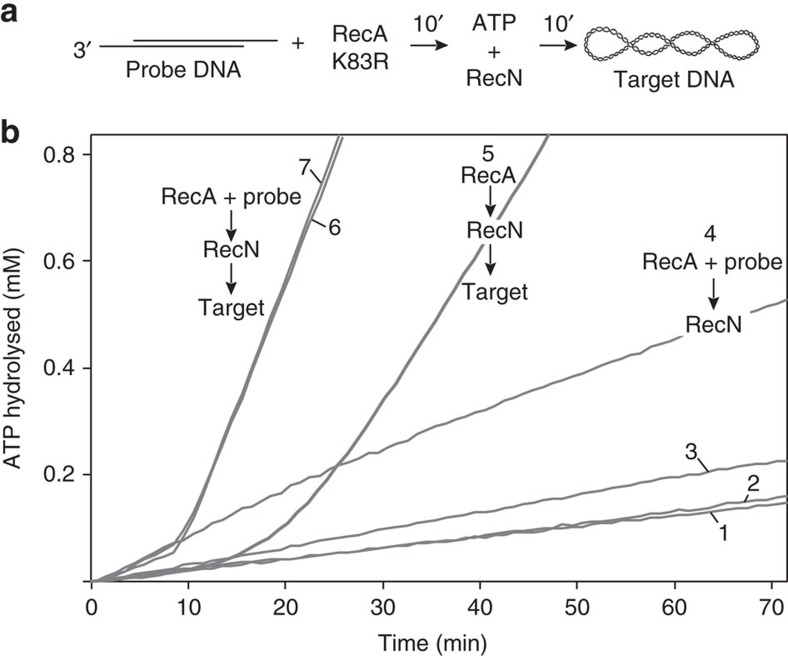
The stimulation of RecN ATPase by RecA protein under D-loop assay conditions is not homology-dependent. (**a**) Schematic of reaction assembly used to monitor RecN ATPase during RecA-dependent D-loop formation. RecA K83R (3.4 μM where indicated) was incubated with probe DNA (see Fig. 2 legend) for 10 min before the addition of 3 mM ATP and RecN (1 μM where indicated). Target DNA (see Fig. 2 legend) was added 10 min later. For each reaction described, components omitted from reactions were compensated for by protein storage buffers or TE, in the case of DNA. All reactions were carried out under buffer A conditions and followed the reaction scheme shown. ATP hydrolysis was measured after the addition of ATP. (**b**) Controls measuring RecN ATP hydrolysis in the absence of RecA K83R are shown with 10 μM probe DNA and no target DNA (reaction 1), no probe DNA and 10 μM target DNA (reaction 2), and 10 μM probe DNA plus 10 μM target DNA (reaction 3). Reaction 4: RecN ATP hydrolysis when RecA K83R protein was incubated with 10 μM probe DNA in the absence of added target DNA. Reaction 5: RecN ATP hydrolysis when RecA K83R protein was incubated in the absence of probe DNA followed by 10 μM target DNA. Reaction 6: RecN ATP hydrolysis when RecA K83R protein was incubated with 10 μM probe DNA followed by 10 μM target DNA. Reaction 7: RecN ATP hydrolysis when RecA K83R protein was incubated with 10 μM probe DNA followed by 10 μM non-homologous, supercoiled RF1 *φ*X174 DNA. See Table 2 for steady-state RecN ATP hydrolysis rates.

To gain insight into the role of ATP hydrolysis, RecN ATPase activity was measured under D-loop assay reaction-optimized conditions (see Methods) using the RecA K83R ATPase-deficient mutant (Fig. 5b). The RecA K83R mutant protein is proficient in mediating D-loop formation, as has been shown for other RecA Walker A mutant homologues (Supplementary Fig. 4 and ref. 24). Under the conditions used, the background RecN ATPase activity is very low (∼2 μM min^−1^, Table 2) in the absence of RecA when the D-loop probe DNA, the target DNA or both DNA substrates (Fig. 5b, reactions 1, 2 and 3, respectively) are included in the reaction. For reactions containing RecA, the DNA and proteins are assembled as indicated in Fig. 5a, and individual reactions in Fig. 5b indicate the protein(s) and the DNA substrate(s) added. Reaction assemblies are also noted in Table 2. The rate of RecN ATP hydrolysis (Table 2) increases in the presence of RecA and either D-loop probe DNA (reaction 4) or D-loop target DNA (reaction 5) and the highest rate is observed when the complete D-loop assay components of both DNA substrates are present (reaction 6). The RecN ATPase rate measured under complete D-loop reaction conditions (reaction 6) represents a 20-fold stimulation (Table 2) compared to the same reaction in the absence of RecA protein (reaction 3). This enhanced rate of RecN ATP hydrolysis under the D-loop reaction conditions is largely dependent on both RecA protein bound to the probe DNA and the presence of the target DNA.

We asked whether the ATPase activity of RecN is dependent on homology. The RecA-dependent D-loop assay was repeated with non-homologous target DNA (supercoiled phage *φ*X174 RF1) and RecN ATPase activity was monitored (reaction 7, Fig. 5b). Interestingly, the measured rate of RecN ATP hydrolysis is the same whether the target DNA is homologous or heterologous to the probe DNA (Table 2). It appears that the enhancement of RecN ATPase observed during the D-loop reaction may not be concomitant with the RecA-mediated DNA-pairing reaction. This suggests that the RecN ATPase activity is required for a presynaptic step of the reaction after RecA protein binds to the probe DNA.

### Target DNA concentration and length affects RecN ATPase

The difference in the measured rate of RecN hydrolysis in RecA–RecN–DNA ternary complexes with the D-loop probe DNA compared to the D-loop target DNA (Table 2) was unexpected (compare reactions 4 and 5, Fig. 5b). While the two DNAs are present at the same concentration (in micromolar nucleotides), the probe DNA contains a 150-nucleotide ssDNA overhang and the target DNA is supercoiled circular duplex DNA. The RecN ATPase rate in the absence of RecA is the same for both of these DNA molecules (reactions 1 and 2, Fig. 5b). We have previously determined that the double-stranded DNA-dependent rate of ATP hydrolysis is similar whether the duplex DNA cofactor is supercoiled, linearized or relaxed circular, even under conditions optimized for RecN ATP hydrolysis^5^. Therefore, we only observe different RecN ATPase rates with the probe DNA and the target DNA when RecA is present in the reaction. A further difference between reactions 4 and 5 of Fig. 5b is the presence of DNA at the time of RecA addition. In reaction 4, RecA is pre-incubated with probe DNA. In reaction 5, RecA is not incubated with DNA before the addition of RecN. Since the RecN hydrolysis rate observed in reaction 5 is much greater than in reaction 4 where RecA is bound to probe DNA, it appears that RecN is not activated for hydrolysis until additional DNA is added to the preformed RecA filaments in the presence of RecN. The only DNA present in reaction 5 is supercoiled DNA and it is possible that the RecA protein is unable to saturate the DNA, so there is free DNA present for RecN to bind to or for RecA/RecN to conduct some unproductive searching. Since the rate of RecN ATP hydrolysis is higher when additional DNA is added as in reaction 6 of Fig. 5b, it is possible that RecN is interacting both with RecA protein bound to probe DNA and to the second DNA strand added (target), as we suggest below in the model of Fig. 7. In reaction 4 of Fig. 5b, the probe DNA (with ssDNA extensions) should be saturated by the RecA protein added and there is much less opportunity to pseudo-search another DNA strand not bound by RecA protein, although there is probably a bit of unbound probe DNA at any point in time. It is possible that to some extent the DNA can act as both probe and target, albeit unproductive due to the four strands present. RecA might not be searching in this scenario, due to the four strands, but RecN may be bringing the two DNA molecules into juxtaposition nonetheless. The idea that RecN–RecA–DNA complexes engaged in unproductive, pseudo-searching also stimulates the RecN ATP hydrolysis is further supported by the fact the two DNAs need not be homologous (as in Fig. 5b, reaction 7).

**Figure 6:**
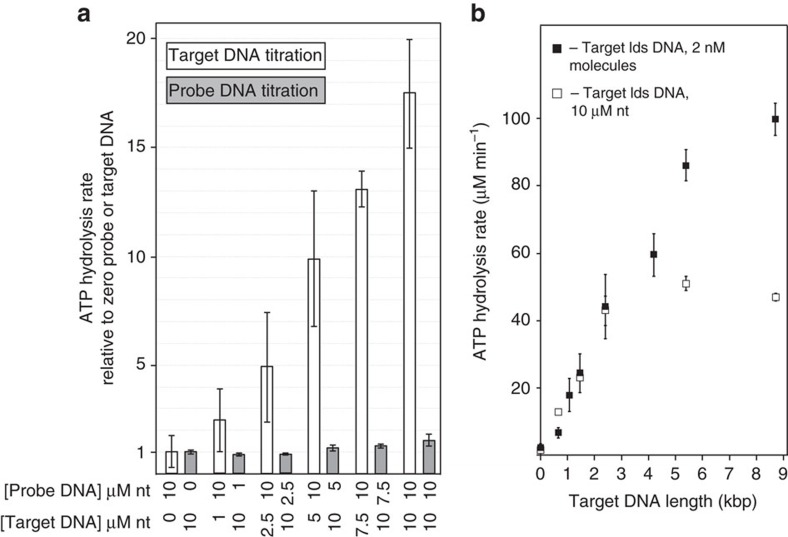
The stimulation of RecN ATPase by RecA protein under D-loop assay conditions is target DNA concentration- and length-dependent. Reactions were carried out in Buffer A and assembled as in reaction 6, including protein concentrations, from Fig. 5b, except as noted. (**a**) The steady-state RecN ATP hydrolysis rate was measured when added to RecA K83R protein incubated with probe DNA (where indicated) followed by target DNA (where indicated). The probe DNA concentration is held constant at 10 μM and the target DNA concentration is titrated (0–10 μM), and white bars represent the rate of ATP hydrolysis catalysed by RecN protein at each DNA concentration relative to the rate measured at zero target DNA. The target DNA concentration is held constant at 10 μM and the probe DNA concentration is titrated (0–10 μM), and grey bars represent the rate of ATP hydrolysis catalysed by RecN protein at each DNA concentration relative to the rate measured at zero probe DNA. Error bars represent the s.d. of the relative rate (the s.d. of the average rate divided by the average rate) from 3 to 15 independent experiments (see Table 2 for steady-state rates). (**b**) The steady-state RecN ATP hydrolysis rate was measured when added to RecA K83R protein incubated with 2 nM molecules (10 μM nt) probe DNA followed by 2 nM molecules linearized, target DNA (target lds DNA, black square) or 10 μM of nucleotides linearized, target DNA (target lds DNA, white square) of different lengths, as indicated. The linear duplex target DNA substrate length in kilobase pairs (kbp) and concentration in μM nt and nM molecules for the two sets are as follows: 0.65 kbp (2.6 μM, black square; 7.7 nM, white square); 1.1 kbp (4.3 μM, black square); 1.5 kbp (5.8 μM, black square; 3.5 nM, white square); 2.4 kbp (9.6 μM, black square; 2.1 nM, white square); 4.2 kbp (16.8 μM, black square); 5.4 bp (21.5 μM, black square; 0.9 nM, white square); and 8.7 kbp (34.8 μM, black square; 0.6 nM, white square). The error bars represent the s.d. of four independent experiments. nt, nucleotide.

To determine whether the stimulation of hydrolysis is indeed related to a second DNA strand addition, we carried out DNA titration experiments in the presence of RecA K83R protein. The D-loop probe DNA concentration was held constant at 10 μM and the D-loop target DNA was titrated from 0 to 10 μM producing a clear dependency of the rate of ATP hydrolysis on the target DNA concentration (Fig. 6a and Table 2) and less concentration dependence on RecN ATPase rates when the probe DNA was titrated.

In addition to titrating the concentration of target DNA as described above, we also investigated the effect of changing the length of the target DNA (Fig. 6b) added to the same D-loop reaction in Figs 5 and 6a. When the concentration of target DNA molecules was held constant at 2 nM (closed squares, Fig. 6b), the RecN ATPase activity measured was proportional to the length of target DNA added to the reaction. Since the concentration of DNA base pairs is also increasing as the DNA length increases (see legend to Fig. 6b), we repeated the DNA target length experiment with a constant concentration of DNA (10 μM) of nucleotides (open squares, Fig. 6b). In this case, the measured RecN ATPase rate with DNA target lengths 2.4 kilobase pairs (kbp) or longer were approximately the same (∼50 μM min^−1^). However, the RecN ATP hydrolysis rate is again higher in the presence of these longer target DNAs than when the shorter target DNAs are added, even though the number of target DNA molecules decreases as the length increases. Again, the RecN ATPase activity measured was proportional to the length of target DNA added to the reaction for DNA lengths below 2.4 kbp.

RecN protein is activated for ATP hydrolysis by RecA bound to DNA (Fig. 4) and the rate of that hydrolysis during a D-loop reaction is largely dependent on the concentration of the target DNA (Fig. 6a). This suggests an activation of RecN protein ATPase where the stimulation occurs as a function of the addition of the second DNA strand (the target) to the RecA protein bound to the probe DNA. The target DNA used in Fig. 5b, reactions 5 and 6 is supercoiled plasmid duplex DNA fully homologous to the probe DNA. We also find that the stimulation of RecN ATP hydrolysis under D-loop conditions (reaction 6, Fig. 5b) is observed even when the target DNA is linearized (Table 2 and Fig. 6b). Therefore, the stimulation of RecN ATPase activity is not unique to a RecA–RecN–supercoiled DNA complex.

## Discussion

We conclude that the SMC-like RecN protein has a presynaptic role in RecA-mediated homologous DNA pairing. We present several lines of evidence suggesting the function of RecN is important after RecA has bound DNA and before the DNA-pairing step. Our results show that (i) RecN stimulates the RecA-dependent DNA strand invasion reaction *in vitro*, (ii) the RecN and RecA proteins physically interact, (iii) the stimulation of RecA requires the ATPase activity of RecN protein, (iv) RecA bound to DNA stimulates the ATPase activity of RecN protein more than 20-fold, (v) the rate of RecN-mediated ATP hydrolysis during the D-loop reaction is sensitive to the concentration and the length of a second DNA strand added (target) and (vi) the stimulation of RecN ATP hydrolysis observed during the D-loop reaction is not homology-dependent.

**Figure 7:**
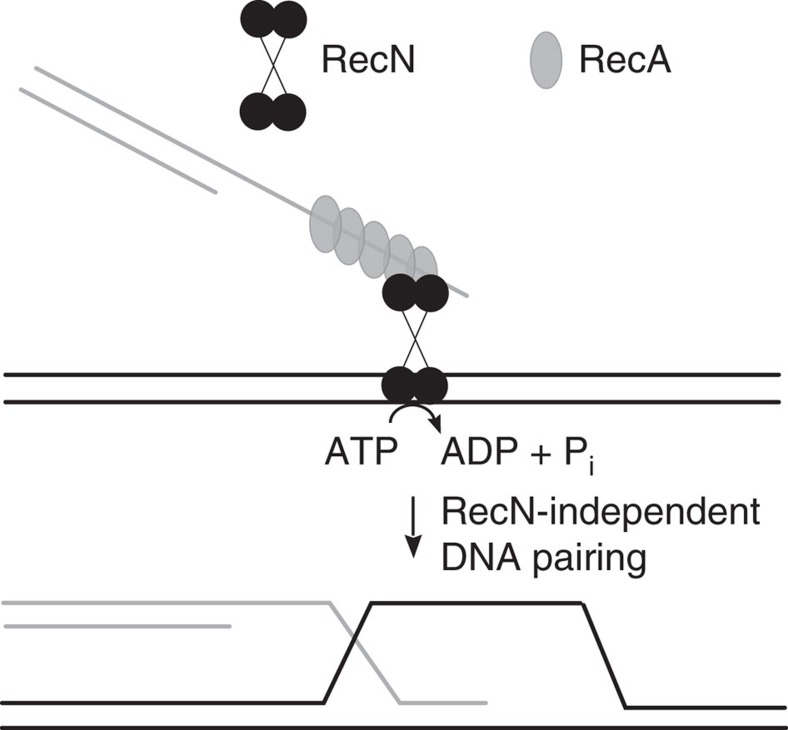
Model for the role of RecN in the stimulation of the RecA strand invasion step of DNA DSB repair. RecN interacts with RecA bound to a ssDNA region of one DNA molecule and with a target duplex DNA molecule. *In vitro*, this scenario leads to a relatively high rate of ATP hydrolysis by the RecN protein. One possible function of RecN ATP usage is the movement of the complex along or between potential target DNA molecules as part of a global search for homology. Alternatively, RecN protein may be affecting RecA–DNA filament dynamics and/or the topological state of the DNA, as discussed in the text.

We propose a role for RecN in the search for homology between two DNA strands, one bound by RecA (Fig. 7). First, RecA binds to a resected DSB end and recruits the RecN protein. We believe the recruitment of RecN is mediated by a direct interaction between RecN and RecA protein. Keyamura *et al*.^9^ has shown that green fluorescent protein-labelled RecN protein fails to localize to the sites of DSBs in the absence of RecA in *E. coli*. Alonso's group has determined the localization kinetics of several *B. subtilis* recombination proteins and reports that RecN is among the first responders to a DSB, followed by the RecA protein^8^,^14^,^25^. Further, the *B. subtilis* RecA protein was shown to promote the disassembly of *B. subtilis* RecN–DNA complexes *in vitro*^20^. In the current study, we demonstrate that an ordered assembly of ternary complex occurs such that RecA binds to DNA followed by the binding of RecN protein (as measured by RecN ATP hydrolysis; Fig. 4). In fact, a measurable lag in the engagement of RecN is observed if RecN is pre-incubated either with the DNA or RecA suggesting a reorganization of proteins is occurring such that RecN is dissociating from DNA or RecA so that RecA is free to bind the DNA.

We have presented several lines of evidence that suggest a direct interaction between RecA and RecN proteins: (i) the stimulation of RecA-mediated DNA pairing and strand exchange by RecN is species-specific (Fig. 3); (ii) the kinetic enhancement of RecN ATP hydrolysis by RecA bound to DNA is species-specific (Supplementary Fig. 3); (iii) the rate of RecN ATP hydrolysis is lower when RecA and RecN are incubated together in the absence of DNA than when RecN is bound to DNA in the absence of RecA (reaction 4, Fig. 4); and (iv) the RecN and RecA proteins co-elute from antibody-coupled resins (Fig. 3).

Once the RecN protein has been recruited to the DNA-bound RecA protein, the ATPase activity of RecN is stimulated (Figs 4 and 5). Monitoring the rates of RecN ATP hydrolysis during the RecA-mediated D-loop reaction allowed us to detect an ordered activation of RecN activity since the stimulation is sensitive to the concentration of the second DNA strand added to the reaction (the target), but is less sensitive to the concentration of the probe DNA bound by RecA protein (Fig. 6a). Further, the increased activity of RecN is likely important for a step of the reaction before the DNA base-level, Watson–Crick sampling that is thought to be the initial steps of homologous pairing^26^,^27^,^28^,^29^ since the rate increase is observed even when the target DNA is not homologous to the probe DNA (Fig. 5b). It is likely that the RecA–DNA–RecN–DNA complex illustrated in Fig. 7 represents an initial bridging of two DNA molecules, a first step in the process of homology search. Although the function(s) of this RecA-dependent increase in ATP hydrolysis by RecN (more than 20-fold under some conditions) is not fully elucidated, the rate increase elicits the intriguing possibility that RecN possesses a motor activity. The high level of ATP hydrolysis by RecN protein indicates that RecN may be affecting the dynamics of RecA-mediated pairing of two DNA strands. It is interesting to note that the eukaryotic Rad54 protein stimulates Rad51 homologous DNA pairing by remodelling DNA topology^30^. RecN could conceivably generate negative topological stress that would stimulate DNA strand invasion by RecA^31^. Although we observe the same activation of RecN ATP hydrolysis whether the target DNA is supercoiled or linearized, a detailed study of the effect of RecN on the topological state of duplex DNA will be required to determine whether this mechanism is a contributing factor.

The ATPase activity of RecN may power movement of RecN or RecN–RecA complexes along the target DNA or facilitate movement between DNA segments. The enhanced rate of RecN ATP hydrolysis under the D-loop reaction conditions (Fig. 5b) is largely dependent on both RecA protein bound to the probe DNA and the concentration of the target DNA. Further, the rate of RecN ATP hydrolysis is sensitive to the length of target DNA (Fig. 6b). This may be a reflection of a higher RecN affinity for longer DNA molecules. RecN likely binds DNA with some degree of cooperativity since the DNA-dependent rate of ATP turnover depends on RecN protein concentration^5^. Although more work is necessary to clearly delineate the mechanism of these effects, another interpretation is that shorter DNA limits the rate of ATP hydrolysis because RecN movement is limited in range on a single DNA-binding event.

Studies by several groups^32^,^33^,^34^ have argued that SMC complexes (both cohesins and condensins) translocate, presumably by sliding, to relocate over large distances along a chromosome (reviewed in refs 35, 36). Therefore, it is possible that RecN translocation is at least contributing to the high rates of ATP hydrolysis we have observed in the current study. In the model proposed in Fig. 7, translocation powered by ATP hydrolysis would allow RecN to move RecA presynaptic filaments (through a direct interaction) along target duplex DNA. At least two previous studies have proposed a three-dimensional diffusion model for the RecA homology search and provide evidence that RecA–DNA filaments do not slide with respect to target duplex DNA^37^,^38^. However, Ragunathan *et al*.^39^ observed one-dimensional sliding of RecA presynaptic filaments with respect to target duplex DNA. It is possible that both sliding and intersegmental transfer contribute to the search for homology^39^,^40^. The RecN movement of RecA presynaptic filaments could contribute to both movement along the chromosome and intersegment transfer between chromosomes as part of a global search for homology in the cell. This idea of a cellular-wide homology search, although not new, is frequently overlooked when describing recombination events^28^. In eukaryotic cells, chromatin mobility increases at sites of DSBs^41^,^42^,^43^, and movement of DNA DSB ends has been visualized in live *E. coli* cells^10^ indicating a presynaptic, long-range homology search in bacterial cells.

The data presented here represent a major step forward in understanding the biochemical role of RecN in recombinational DNA DSB repair pathways. To the best of our knowledge, this study is the first observation of a RecN protein stimulating the DNA strand exchange activity (Fig. 1), and in particular, the formation of D-loops catalysed by the RecA protein (Fig. 2). Further, we have provided evidence for a role of RecN ATP hydrolysis during the presynaptic steps of DSB repair. The elucidation of the bacterial RecN protein activities may further the understanding of recombinational DNA repair in eukaryotic cells. The enhancement of the DNA-pairing activity of RecA by the RecN protein is strikingly similar to the stimulation of Rad51 by the Rad54 protein or of Dmc1 by Hop2–Mnd1 proteins shown by several groups^44^,^45^,^46^,^47^,^48^,^49^,^50^,^51^,^52^. The activity of RecN as a first responder to DSBs has also been compared to the Rad50 component of the Mre11–Rad50–Nbs1 complex^14^,^53^. The model presented in Fig. 7 supposes a direct link between the assembly of RecN and RecA at a DNA DSB and the RecN facilitation of a long-range homology search by RecA protein that leads to the critical homologous DNA-pairing event of the recombinational DNA repair pathway.

## Methods

### Protein expression and purification

*Wild-type RecN protein*. The *D. radiodurans* RecN protein was expressed in EAW3 (*E. coli* strain MG1655 *ΔrecN*; ref. 5) cells co-transformed with pT7Pol26 and pEAW309 (ref. 5). A 10 l culture was grown in Luria broth (LB) broth (10 g l^−1^ tryptone, 5 g l^−1^ yeast extract and 10 g l^−1^ NaCl, with pH adjusted to 7.0) to an OD_600_ of 0.5. RecN protein expression was induced by the addition of isopropyl β-D-1-thiogalactopyranoside (IPTG) to a final concentration of 0.4 mM. Following a 6 h incubation at 37 °C, cells were collected by centrifugation, flash-frozen in liquid N_2_ and stored at −80 °C. All subsequent steps were carried out at 4 °C. Cell paste was thawed and fully resuspended to 20% cell weight per volume ratio in Tris-sucrose solution (25% sucrose and 250 mM Tris-HCl 80% cation, pH 7.5) supplemented with the protease inhibitors 4-(2-aminoethyl)benzenesulfonyl fluoride-HCl and pepstatin A to final concentrations of 0.5 mg ml^−1^ and 0.7 μg ml^−1^, respectively. The cells were lysed by 60 min incubation with lysozyme in 250 mM Tris-HCl (80% cation, pH 7.5) to 2.5 mg ml^−1^, followed by the addition of 0.4 ml of 25 mM EDTA per ml of lysed cell suspension, sonication and centrifugation for 1 h. The lysate was precipitated with polyethyleneimine, pH 7.5 (0.5% final concentration) and centrifuged. The pellet was washed with R buffer (20 mM Tris-HCl (80% cation, pH 7.5), 1 mM dithiothreitol (DTT), 0.1 mM EDTA and 10% glycerol)+150 mM ammonium sulfate and extracted two times with R buffer+300 mM ammonium sulfate. The protein solution was precipitated by the addition of 0.23 g solid ammonium sulfate per ml of solution (40% saturation). The precipitant was washed with R buffer+2.3 M ammonium sulfate, resuspended in R buffer+300 mM KCl and dialysed extensively versus R buffer+50 mM KCl. The protein was loaded onto DEAE-Sepharose resin (GE Healthcare), washed with 1 column volume of R buffer+50 mM KCl and eluted with a linear gradient of KCl from 50 mM to 1 M KCl over 10 column volumes. The RecN protein eluted at ∼300 mM KCl. Peak fractions were analysed by SDS–PAGE and fractions containing RecN were pooled and dialysed versus P buffer (20 mM potassium phosphate (pH 6.8), 1 mM DTT, 0.1 mM EDTA and 10% glycerol). Pooled protein was loaded onto ceramic hydroxyapatite resin (BioRad), washed with 1 column volume of P buffer and eluted with a linear gradient from 20 mM to 1 M potassium phosphate buffer (pH 6.8) over 10 column volumes. The RecN protein eluted at ∼ 400 mM phosphate. The fractions containing RecN were pooled and concentrated using a Source Q column. The pure RecN protein was determined to be free of nuclease contamination and was dialysed extensively versus storage buffer (R buffer+50 mM KCl), flash-frozen in liquid N_2_ and stored at −80 °C. The concentration of the RecN protein (molecular weight 59,798 Da) was determined from the absorbance at 280 nm using the calculated extinction coefficient 29,160 M^−1^ cm^−1^.

*RecN K67A mutant protein*. Cloning of pPLP01 containing *D. radiodurans recN K67A* was carried out via site-directed mutagenesis, using pEAW309 (wild-type *recN* expression plasmid^5^) as a template, according to the Stratagene Quick Change site-directed mutagenesis kit manual. DNA sequencing confirmed the desired point mutation. The *D. radiodurans* RecN K67A mutant protein was expressed and purified as described above for wild-type RecN protein except that the protein required flow through passage in Heparin FF resin (GE Healthcare) to remove trace nuclease contamination before the final concentration step.

*D. radiodurans RecA proteins*. The wild-type RecA and the RecA K83R mutant proteins were purified following a modified version of a procedure previously described^54^. Briefly, the *D. radiodurans* wild-type RecA or RecAK83R proteins were expressed by growing a 10 l culture of *E. coli* strain STL2669 (ref. 55) co-transformed with either pEAW158, (wild-type *recA*) or pEAW244 (*recA K83R*) and pT7Pol26 in LB broth to an OD_600_ of 0.6. The pEAW158 and pEAW244 expression plasmids were gifts from Michael Cox (University of Wisconsin-Madison). Expression was induced by addition of IPTG to a final concentration of 0.4 mM. Following a 3 hour incubation with IPTG at 37 °C, the cells were collected by centrifugation, flash-frozen in liquid N_2_ and stored at −80 °C. All subsequent steps of this purification were carried out at 4 °C. Cells were thawed and fully resuspended to a final 20% cell weight per volume ratio in Tris-sucrose solution. Cell suspensions were lysed by 60 min incubation with lysozyme in 250 mM Tris-HCl (80% cation, pH 7.5) to 2.5 mg ml^−1^, followed by the addition of 0.4 ml of 25 mM EDTA per ml of lysed cell suspension, sonication and centrifugation for 1 h. The lysates were precipitated with polyethyleneimine (Sigma), pH 7.5 (0.5% final concentration) and centrifuged. The resulting pellets were washed with 50 ml of R buffer+50 mM ammonium sulfate and then extracted two times with 50 ml of R buffer+300 mM ammonium sulfate. The protein solutions were precipitated by adding 0.33 g solid ammonium sulfate per ml of solution (55% saturation). The precipitants were washed with 50 ml R buffer+3 M ammonium sulfate, resuspended in 50 ml of R buffer+300 mM KCl and dialysed versus R buffer+150 mM KCl and then extensively dialysed into R buffer+50 mM KCl. The proteins were loaded onto a DEAE-Sepharose column and washed with two column volumes of R buffer+50 mM KCl. Flow-through fractions were identified by SDS–PAGE, pooled and dialysed into P buffer. The dialysed proteins were then loaded onto Bio-Gel hydroxyapatite resin (BioRad), washed with two column volumes of P buffer and eluted with two column volumes of 500 mM potassium phosphate buffer (pH 6.8). Pooled fractions were dialysed verses R buffer+50 mM KCl and loaded onto a PBE-94 column (GE Healthcare), washed with one column volume of R buffer+50 mM KCl and eluted with a linear gradient from 50 mM to 1 M KCl. The RecA proteins eluted from this column at ∼500 mM KCl. Pooled protein fractions were determined to be free of nuclease contamination and were concentrated by ammonium sulfate precipitation at 55% saturation as described above. Pellets were resuspended in 5 ml of R buffer+300 mM KCl and dialysed extensively into R buffer (storage buffer). Concentrations of *D. radiodurans* RecA proteins were determined from the absorbance at 280 nm, using the determined extinction coefficient 0.372 mg ml^−1^ cm^−1^ and molecular mass 38,013 Da (ref. 54).

*E. coli RecA protein*. The *E. coli* wild-type RecA protein was purified as described^55^. Briefly, the RecA protein was overexpressed by growing a 10 l culture of STL2669 (ref. 5) co-transformed with pAIR79 (wild-type *recA*) and pT7pol26 in LB broth to an OD_600_ of 0.8. Protein expression was induced by the addition of IPTG to a final concentration of 0.4 mM. Following a 3 h incubation with IPTG at 37 °C, the cells were collected by centrifugation, flash-frozen in liquid N_2_ and stored at −80 °C. All subsequent steps of this purification were carried out at 4 °C. Cell paste was thawed and fully resuspended to a final 20% cell weight by volume ratio in Tris-sucrose solution. Cell suspension was lysed by a 60 min incubation with lysozyme (2.5 mg ml^−1^ final) in 250 mM Tris-HCl (80% cation, pH 7.5) followed by the addition of 0.4 ml of 25 mM EDTA per ml of fraction, sonication and centrifugation. The lysate was precipitated with polyethyleneimine, pH 7.5 (0.5% final concentration) and centrifuged. The pellet was washed once with 100 ml of R buffer+150 mM ammonium sulfate and then extracted two times with 100 ml of R buffer+300 mM ammonium sulfate. The protein solution was precipitated by adding 0.28 g of solid ammonium sulfate per ml of solution (47% saturation) followed by centrifugation. The precipitated protein was washed twice with 50 ml R buffer+2.8 M ammonium sulfate, resuspended in 100 ml of R buffer+100 mM KCl and dialysed extensively into R buffer+100 mM KCl. The protein was loaded onto DEAE-Sepharose resin and washed with two column volumes of R buffer+100 mM KCl. Flow-through fractions were identified by SDS–PAGE, pooled and dialysed into P buffer and loaded onto ceramic hydroxyapatite resin, washed with two column volumes of P buffer. Protein was eluted with a linear gradient from 20 to 350 mM potassium phosphate over 10 column volumes. Pooled protein fractions were determined to be free of nuclease contamination and were concentrated by ammonium sulfate precipitation at 47% saturation as described above, resuspended in R buffer and dialysed extensively into R buffer (storage buffer). The concentration of purified RecA protein (37,842 Da) was determined from the absorbance at 280 nm using the extinction coefficient 2.23 × 10^4^ M^−0^ cm^−c^ (ref. 55).

*Single-strand binding proteins*. The *E. coli* single-strand binding (SSB) protein was purified as described^56^. Briefly, the *E. coli* SSB protein was overexpressed by growing a 10 l culture of BL21(DE3) transformed with pEAW134 (a gift from Michael Cox) in LB broth to an OD_600_ of 0.5. Protein expression was induced by the addition of IPTG to a final concentration of 0.4 mM. Following a 3 h incubation with IPTG at 37 °C, the cells were collected by centrifugation and flash-frozen in liquid N_2_. All steps of this purification were carried out at 4 °C. Cell paste was thawed and fully resuspended to a final 25% cell weight to volume ratio in lysis buffer (50 mM Tris-HCl, pH 8.3, 0.2 M NaCl, 15 mM spermidine tri-Cl, 1 mM EDTA and 10% sucrose). Cell suspension was lysed by a 30 min incubation with lysozyme (200 μg ml^−1^ final lysozyme concentration). The cell suspension was also supplemented with phenylmethylsulfonyl fluoride (0.1 mM final concentration). Cell lysate was incubated for 30 additional minutes with sodium deoxycholate (0.05% final concentration) followed by sonication and centrifugation. The lysate was precipitated with polyethyleneimine, pH 7.5 (0.4% final concentration) for 30 min and centrifuged. The pellet was resuspended for 45 min in 100 ml of TGE buffer (50 mM Tris-HCl, pH 8.3, 1 mM EDTA and 20% glycerol)+0.4 M NaCl and centrifuged. The protein solution was supplemented with 0.15 g ml^−1^ ammonium sulfate (27% saturation) overnight and centrifuged. The precipitant was washed in 100 ml of TGE buffer+0.15 g ml^−1^ ammonium sulfate and centrifuged. Washing step was repeated twice. Protein pellet was resuspended in 20 ml of TGE buffer+300 mM NaCl and subjected to step dialysis into TGE buffer+50 mM NaCl. The solution was loaded onto DEAE-Sepharose resin, washed with 2 column volumes of TGE buffer+50 mM NaCl and eluted with a linear gradient from 50 to 800 mM NaCl over 10 column volumes. Pooled fractions were dialysed into TGE buffer+50 mM NaCl, loaded onto Heparin resin, washed with 2 column volumes of TGE buffer+50 mM NaCl and eluted with a linear gradient from 50 to 300 mM NaCl over 10 column volumes. Pooled fractions were determined to be free from nuclease contamination and dialysed extensively into storage buffer (20 mM Tris-HCl, pH 8.3, 0.5 M NaCl, 1 mM EDTA, 1 mM β-mercaptoethanol and 50% glycerol). The concentrations of *E. coli* SSB (18,843 Da) was determined by absorbance measurements at 280 nm using the extinction coefficient of 2.38 × 10^4^ M^−1^ cm^−1^ (ref. 56). The purified *D. radiodurans* SSB protein^57^ was a gift from Michael Cox.

### Biochemicals

Unless otherwise noted, all of the reagents were purchased from Fisher. Phosphoenolpyruvate was from Spectrum. Pyruvate kinase, lactate dehydrogenase, NADH, ATP, polyethyleneimine and bromophenol blue were purchased from Sigma. All restriction endonucleases and T7 exonuclease were purchased from New England Biolabs. DTT was from Soltec Ventures.

### DNA substrates

Bacteriophage *φ*X174 circular ssDNA (virion) and *φ*X174 RFI supercoiled circular duplex DNA (5,386 bp) were purchased from New England Biolabs. Plasmid DNA substrates pEAW324 (8,716 bp) and pEAW3 (2,431 bp) were gifts from Michael Cox^58^,^59^, and prepared using CsCl-ethidium bromide gradients^60^. Unless otherwise noted, full-length linear duplex DNA substrates were generated by the digestion at unique restriction sites on the *φ*X174 RFI DNA with PstI, pEAW324 with ApaI and pEAW3 with SspI restriction endonucleases, using conditions suggested by the enzyme supplier. The digested DNA was extracted with phenol/chloroform/isoamyl alcohol (25:24:1), followed by ethanol precipitation. D-loop substrate described as target DNA is supercoiled pEAW3 plasmid, unless otherwise indicated. D-loop substrate described as probe DNA (Figs 2a and 5) was prepared by incubation of SspI treated pEAW3 linear duplex DNA with T7 exonuclease at 30 °C for 90 s. T7 exonuclease catalyses the removal of 5′-mononucleotides from duplex DNA in the 5′–3′ direction at a rate of ∼150 nucleotides per 90 s in buffer conditions suggested by enzyme supplier. Various-length linear duplex DNA substrates used as target for the experiments of Fig. 6b were prepared by digesting pEAW3 (2,431 bp) with NdeI, XbaI and ScaI to produce 1,450, 650 and 303 bp and pEAW324 (8,716 bp) with BsaXI to produce 4,205 and 1,074 bp, using conditions suggested by the enzyme supplier. DNA substrates were gel-purified using Agarase (Thermo Scientific) following the protocol for recovery of DNA from low-melting agarose gels provided by the supplier. The concentrations of ssDNA and double-stranded DNA were determined by absorbance at 260 nm, using 36 and 50 mg ml^−1^ A_260_^−1^, respectively, as conversion factors. Unless otherwise specified, all DNA concentrations are given in micromolar nucleotides.

### Buffers

Buffer A is 25 mM Tris acetate (80% cation, pH 7.4), 1 mM DTT, 3 mM potassium glutamate, 10 mM Mg(OAc)_2_ and 5% (weight per volume) glycerol. Buffer N is 25 mM Tris-OAc (80% cation, pH 7.4), 1 mM DTT, 3 mM potassium glutamate, 17.5 mM Mg(OAc)_2_, 40 mM KOAc, 5% (weight per volume) glycerol and 1% buffered polyethylene glycol 8000. Buffer T is 20 mM Tris-Cl (80% cation, pH 7.4), 20 mM EDTA. 0.5% SDS. 2 × loading buffer contains 15% Ficoll, 0.24% bromphenol blue and 0.24% xylene cyanole. 2 × loading stop buffer solution contains 15% Ficoll, 4% SDS, 0.24% bromophenol blue and 0.24% xylene cyanole. TBE buffer is 90 mM Tris borate and 2 mM EDTA, pH 8. 5 × SDS loading buffer contains 250 mM Tris-Cl (pH 6.8), 4% SDS, 20% glycerol, 10% 2-mercaptoethanol and 0.1% bromophenol blue.

### RecA-mediated DNA three-strand exchange

The DNA strand exchange reactions were carried out at 37 °C in buffer A and an ATP regeneration system (10 units per ml of pyruvate kinase and 2.5 mM phosphoenolpyruvate). Protein and DNA concentrations are described in figure legends. Experiments measuring the DNA strand exchange activity of the *E. coli* or *D. radiodurans* RecA protein utilized the *E. coli* or *D. radiodurans* SSB protein, respectively. RecA protein was incubated with *φ*X174 circular ssDNA for 10 min. SSB protein, 3 mM ATP and the RecN protein (where indicated) were added, followed by another 10 min incubation. The reaction was initiated by the addition of *φ*X174 linear duplex DNA and incubated for 45 min, or the times indicated in the figure legend. Dilute condition reactions (Figs 1c and 3a) were stopped by addition of 80 μl buffer T plus 5 μl of proteinase K (to 1.25 mg ml^−1^). DNA was extracted with phenol/chloroform/isoamyl alcohol (25:24:1), followed by ethanol precipitation. DNA was resuspended in TE (10 μl) plus 2 × loading buffer. Normal DNA concentration reactions (Fig. 1b) were stopped by adding 2 × loading stop buffer and proteinase K (to 1.25 mg ml^−1^). Samples were subjected to electrophoresis in 0.8% agarose gels with TBE buffer, stained with ethidium bromide and exposed to ultraviolet light. The inverted gel images were obtained using a digital charge-coupled device camera with Foto/Analyst PC Image software version 10.21 (Fotodyne).

### RecA-dependent D-loop formation

The D-loop formation reactions were carried out at 37 °C in buffer A and an ATP regeneration system (10 units per ml of pyruvate kinase and 2.5 mM phosphoenolpyruvate). Protein and DNA concentrations are described in figure legends. RecA protein was incubated with probe DNA for 10 min. ATP (3 mM) and the RecN protein were added, followed by another 10 min incubation. The reaction was initiated by the addition of target DNA and incubated for 45 min. Reactions were stopped by adding 2 × loading stop buffer and proteinase K (to 1.25 mg ml^−1^). Samples were subjected to electrophoresis as described above.

### ATPase assay

The ATPase activity of RecN protein was measured using an enzyme-coupled spectrophotometric enzyme assay as described^5^. All reactions were carried out at 37 °C. The RecN ATPase activity measured in experiments represented in Figs 5 and 6, and were carried out in buffer A. Buffer N was used in experiments represented in Fig. 4. The concentration of proteins and DNA, RecN, as well as the order of protein additions are indicated in the figure legends. Reactions were initiated by the addition of 3 mM ATP.

### Co-elution of RecA and RecN proteins

Pull-down experiments were done using purified *D. radiodurans* RecA and RecN proteins. Antibodies against these proteins were raised in chicken (Chicken IgY) and affinity-purified (GeneTel Laboratories, LLC, Madison, WI). The stock concentration for the RecA and RecN antibodies are 13.2 and 1 mg ml^−1^, respectively. A unit of 50 μg antibody for RecA or RecN (diluted directly from the stock in AminoLink Plus (Pierce) coupling buffer) was coupled to 100 μl of AminoLink Plus coupling resin (50% slurry). All concentrations given are final concentrations. Reactions (40 μl) were carried out in buffer N with an ATP regeneration system (10 units per ml pyruvate kinase and 3.5 mM phosphoenolpyruvate) in the presence or absence of 25 μM *φ*X174 3′-linear duplex DNA. RecA protein (2 μM final) was added to the above reaction mixture and incubated at 37 °C for 20 min. Binding reactions were initiated by the addition of ATP (to 2.5 mM) and allowed to proceed for 20 min before the addition of 4.8 μg RecN protein (2 μM final) and further incubated for 15 min at 37 °C. Samples (except for the input sample) were diluted 1:3 in 1 × Dulbecco's modified PBS buffer (Sigma). Diluted mixtures were loaded on RecA or RecN antibody-coupled resin and incubated for 2 h at 4 °C on a turn-table. Resins were washed with 150 μl IP lysis/wash buffer (Pierce). Protein complexes were eluted with 50 μl of elution buffer (Pierce). Protein samples were mixed directly with 5 × SDS loading buffer, and 10 μl were loaded and separated by 14% SDS–PAGE. The inverted gel images were obtained using a digital charge-coupled device camera with Foto/Analyst PC Image software version 10.21 (Fotodyne).

### Data availability

All relevant data are available from the authors.

## Additional information

**How to cite this article:** Uranga, L. A. *et al*. The cohesin-like RecN protein stimulates RecA-mediated recombinational repair of DNA double-strand breaks. *Nat. Commun.* **8,** 15282 doi: 10.1038/ncomms15282 (2017).

**Publisher's note**: Springer Nature remains neutral with regard to jurisdictional claims in published maps and institutional affiliations.

## Supplementary Material

Supplementary Information Supplementary figures and supplementary references.

## Figures and Tables

**Table 1:** Kinetic measurements for experiments illustrated in Fig. 4.

**Order of addition**	**Average ATP hydrolysis rate (μM min**^**−1**^**)±s.d.**	**Average lag time (min)±s.d.**
1—RecN, no DNA, no RecA K83R	8.3±1.7	0
2—RecN+DNA, no RecA K83R	13.7±4.1	0
3—RecN+DNA→RecA K83R	53.4±4.2	25.0±5.0
4—RecN+RecA K83R→DNA	47.5±8.5	34.3±5.3
5—RecA K83R+DNA→RecN	56.3±15.6	8.3±6.7
RecA K83R+DNA, no RecN	0.20±0.17	0

RecN ATP hydrolysis rate (in μM min^−1^) and lag times (in min) for the experiments illustrated in Fig. 4. The order of additions match the numbered reactions. The ATP hydrolysis rates were measured after the reaction reached the steady state. Lag times, where applicable, represent the time required for the reaction to reach a steady-state rate of hydrolysis after the addition of all reaction components (Fig. 4). All averages and s.d.'s were calculated from 4 independent trials except that data for reaction 5 were calculated from 10 independent trials.

**Table 2:** Steady-state RecN ATP hydrolysis rates under D-loop assay conditions.

**Order of addition**	***n***	**Probe DNA (μM nt)**	**Target DNA (μM nt)**	**Average ATP hydrolysis rate (μM min**^**−1**^**)±s.d.**
RecN+target DNA control (reaction 1, Fig. 5b)	3	0	10	2.4±0.5
RecN+probe DNA control (reaction 2, Fig. 5b)	3	10	0	2.4±0.4
RecN+target and probe DNA control (reaction 3, Fig. 5b)	3	10	10	2.3±0.5
RecA K83R+probe DNA→RecN (reaction 4, Figs 5b and 6a)	9	10	0	4.5±1.8
RecA K83R→RecN+target DNA (reaction 5, Figs 5b and 6a)	9	0	10	30.9±3.2
RecA K83R+probe DNA→RecN+target DNA (Fig. 6a)	5	1	10	27.3±1.7
	8	2.5	10	28.5±1.7
	5	5	10	36.1±4.2
	3	7.5	10	39.7±2.4
	5	10	1	7.7±4.7
	8	10	2.5	14.5±7.4
	3	10	5	29.2±9.3
	5	10	7.5	38.6±2.5
RecA K83R+probe DNA→RecN+target DNA (reaction 6, Figs 5b and 6a)	15	10	10	49.2±6.4
RecA K83R+probe DNA→RecN+heterologous target DNA (reaction 7, Fig. 5b)	3	10	10	48.3±1.4
RecA K83R+probe DNA→RecN+linearized, homologous target DNA (Fig. 6b, 2.4 kb target DNA)	6	10	10	47.9±5.0

Reaction conditions are described in the legend to Fig. 5. Averages and s.d.'s were calculated from the number of independent trials indicated (*n*). Order of addition describes the experimental condition and the relevant figure containing representative data. The probe and target DNAs are derived from 2.4 kbp plasmid DNA and are described in the legend to Fig. 2. Heterologous DNA is non-homologous, supercoiled RF1 *φ*X174 DNA.

## References

[b1] Mehta A. & Haber J. E. Sources of DNA double-strand breaks and models of recombinational DNA repair. Cold Spring Harb. Perspect. Biol. 6, a016428 (2014). 25104768 10.1101/cshperspect.a016428 PMC4142968

[b2] Li X. & Heyer W. D. Homologous recombination in DNA repair and DNA damage tolerance. Cell Res. 18, 99–113 (2008). 18166982 10.1038/cr.2008.1 PMC3087377

[b3] Lusetti S. L. & Cox M. M. The bacterial RecA protein and the recombinational DNA repair of stalled replication forks. Annu. Rev. Biochem. 71, 71–100 (2002). 12045091 10.1146/annurev.biochem.71.083101.133940

[b4] Hirano T. At the heart of the chromosome: SMC proteins in action. Nat. Rev. Mol. Cell Biol. 7, 311–322 (2006). 16633335 10.1038/nrm1909

[b5] Reyes E. D., Patidar P. L., Uranga L. A., Bortoletto A. S. & Lusetti S. L. RecN is a cohesin-like protein that stimulates intermolecular DNA interactions *in vitro*. J. Biol. Chem. 285, 16521–16529 (2010). 20360008 10.1074/jbc.M110.119164 PMC2878014

[b6] Pellegrino S. . Structural and functional characterization of an SMC-like protein RecN: new insights into double-strand break repair. Structure 20, 2076–2089 (2012). 23085075 10.1016/j.str.2012.09.010

[b7] Lloyd R. G., Picksley S. M. & Prescott C. Inducible expression of a gene specific to the RecF pathway for recombination in *Escherichia coli* K12. Mol. Gen. Genet. 190, 162–167 (1983). 6343801 10.1007/BF00330340

[b8] Sanchez H., Kidane D., Castillo Cozar M., Graumann P. L. & Alonso J. C. Recruitment of *Bacillus subtilis* RecN to DNA double-strand breaks in the absence of DNA end processing. J. Bacteriol. 188, 353–360 (2006). 16385024 10.1128/JB.188.2.353-360.2006 PMC1347269

[b9] Keyamura K., Sakaguchi C., Kubota Y., Niki H. & Hishida T. RecA protein recruits structural maintenance of chromosomes (SMC)-like RecN protein to DNA double-strand breaks. J. Biol. Chem. 288, 29229–29237 (2013). 23974212 10.1074/jbc.M113.485474 PMC3795224

[b10] Lesterlin C., Ball G., Schermelleh L. & Sherratt D. J. RecA bundles mediate homology pairing between distant sisters during DNA break repair. Nature 506, 249–253 (2014). 24362571 10.1038/nature12868 PMC3925069

[b11] Meddows T. R., Savory A. P., Grove J. I., Moore T. & Lloyd R. G. RecN protein and transcription factor DksA combine to promote faithful recombinational repair of DNA double-strand breaks. Mol. Microbiol. 57, 97–110 (2005). 15948952 10.1111/j.1365-2958.2005.04677.x

[b12] Picksley S. M., Attfield P. V. & Lloyd R. G. Repair of DNA double-strand breaks in *Escherichia coli* K12 requires a functional *recN* product. Mol. Gen. Genet. 195, 267–274 (1984). 6092851 10.1007/BF00332758

[b13] Sargentini N. J. & Smith K. C. Quantitation of the involvement of the *recA, recB, recC, recF, recJ, recN, lexA, radA, radB, uvrD*, and *umuC* genes in the repair of X-ray-induced DNA double-strand breaks in *Escherichia coli*. Radiat. Res. 107, 58–72 (1986). 3526390

[b14] Alonso J. C. . Early steps of double-strand break repair in *Bacillus subtilis*. DNA Repair 12, 162–176 (2013). 23380520 10.1016/j.dnarep.2012.12.005

[b15] Ayora S. . Double-strand break repair in bacteria: a view from *Bacillus subtilis*. FEMS Microbiol. Rev. 35, 1055–1081 (2011). 21517913 10.1111/j.1574-6976.2011.00272.x

[b16] Odsbu I. & Skarstad K. DNA compaction in the early part of the SOS response is dependent on RecN and RecA. Microbiology 160, 872–882 (2014). 24615185 10.1099/mic.0.075051-0

[b17] Nagashima K. . Degradation of *Escherichia coli* RecN aggregates by ClpXP protease and its implications for DNA damage tolerance. J. Biol. Chem. 281, 30941–30946 (2006). 16914543 10.1074/jbc.M606566200

[b18] Neher S. B. . Proteomic profiling of ClpXP substrates after DNA damage reveals extensive instability within SOS regulon. Mol. Cell 22, 193–204 (2006). 16630889 10.1016/j.molcel.2006.03.007

[b19] Sanchez H., Carrasco B., Cozar M. C. & Alonso J. C. *Bacillus subtilis* RecG branch migration translocase is required for DNA repair and chromosomal segregation. Mol. Microbiol. 65, 920–935 (2007). 17640277 10.1111/j.1365-2958.2007.05835.x

[b20] Sanchez H., Cardenas P. P., Yoshimura S. H., Takeyasu K. & Alonso J. C. Dynamic structures of *Bacillus subtilis* RecN-DNA complexes. Nucleic Acids Res. 36, 110–120 (2008). 17999999 10.1093/nar/gkm759 PMC2248758

[b21] Kim J. I. & Cox M. M. The RecA proteins of *Deinococcus radiodurans* and *Escherichia coli* promote DNA strand exchange via inverse pathways. Proc. Natl Acad. Sci. USA 99, 7917–7921 (2002). 12048253 10.1073/pnas.122218499 PMC122995

[b22] Warfel J. D. & LiCata V. J. Enhanced DNA binding affinity of RecA protein from *Deinococcus radiodurans*. DNA Repair 31, 91–96 (2015). 26021744 10.1016/j.dnarep.2015.05.002

[b23] Grove J. I., Wood S. R., Briggs G. S., Oldham N. J. & Lloyd R. G. A soluble RecN homologue provides means for biochemical and genetic analysis of DNA double-strand break repair in *Escherichia coli*. DNA Repair 8, 1434–1443 (2009). 19846353 10.1016/j.dnarep.2009.09.015

[b24] Rehrauer W. M. & Kowalczykowski S. C. Alteration of the nucleoside triphosphate (NTP) catalytic domain within *Escherichia coli* recA protein attenuates NTP hydrolysis but not joint molecule formation. J. Biol. Chem. 268, 1292–1297 (1993). 8419331

[b25] Kidane D., Sanchez H., Alonso J. C. & Graumann P. L. Visualization of DNA double-strand break repair in live bacteria reveals dynamic recruitment of *Bacillus subtilis* RecF, RecO and RecN proteins to distinct sites on the nucleoids. Mol. Microbiol. 52, 1627–1639 (2004). 15186413 10.1111/j.1365-2958.2004.04102.x

[b26] Howard-Flanders P., West S. C. & Stasiak A. Role of RecA protein spiral filaments in genetic recombination. Nature 309, 215–219 (1984). 6325943 10.1038/309215a0

[b27] Gupta R. C., Folta-Stogniew E., O'Malley S., Takahashi M. & Radding C. M. Rapid exchange of A:T base pairs is essential for recognition of DNA homology by human Rad51 recombination protein. Mol. Cell 4, 705–714 (1999). 10619018 10.1016/s1097-2765(00)80381-0

[b28] Renkawitz J., Lademann C. A. & Jentsch S. Mechanisms and principles of homology search during recombination. Nat. Rev. Mol. Cell Biol. 15, 369–383 (2014). 24824069 10.1038/nrm3805

[b29] Spies M. There and back again: new single-molecule insights in the motion of DNA repair proteins. Curr. Opin. Struct. Biol. 23, 154–160 (2013). 23260129 10.1016/j.sbi.2012.11.008

[b30] Van Komen S., Petukhova G., Sigurdsson S., Stratton S. & Sung P. Superhelicity-driven homologous DNA pairing by yeast recombination factors Rad51 and Rad54. Mol. Cell 6, 563–572 (2000). 11030336 10.1016/s1097-2765(00)00055-1

[b31] Shibata T., Ohtani T., Chang P. K. & Ando T. Role of superhelicity in homologous pairing of DNA molecules promoted by *Escherichia coli* recA protein. J. Biol. Chem. 257, 370–376 (1982). 7031062

[b32] Hu B. . ATP hydrolysis is required for relocating cohesin from sites occupied by its Scc2/4 loading complex. Curr. Biol. 21, 12–24 (2011). 21185190 10.1016/j.cub.2010.12.004 PMC4763544

[b33] Minnen A. . Control of Smc coiled coil architecture by the ATPase heads facilitates targeting to chromosomal parb/pars and release onto flanking DNA. Cell Rep. 14, 2003–2016 (2016). 26904953 10.1016/j.celrep.2016.01.066 PMC4785775

[b34] Wang X. . Condensin promotes the juxtaposition of DNA flanking its loading site in *Bacillus subtilis*. Genes Dev. 29, 1661–1675 (2015). 26253537 10.1101/gad.265876.115 PMC4536313

[b35] Ocampo-Hafalla M. T. & Uhlmann F. Cohesin loading and sliding. J. Cell Sci. 124, 685–691 (2011). 21321326 10.1242/jcs.073866

[b36] Uhlmann F. SMC complexes: from DNA to chromosomes. Nat. Rev. Mol. Cell Biol. 17, 399–412 (2016). 27075410 10.1038/nrm.2016.30

[b37] Adzuma K. No sliding during homology search by RecA protein. J. Biol. Chem. 273, 31565–31573 (1998). 9813072 10.1074/jbc.273.47.31565

[b38] Forget A. L. & Kowalczykowski S. C. Single-molecule imaging of DNA pairing by RecA reveals a three-dimensional homology search. Nature 482, 423–427 (2012). 22318518 10.1038/nature10782 PMC3288143

[b39] Ragunathan K., Liu C. & Ha T. RecA filament sliding on DNA facilitates homology search. Elife 1, e00067 (2012). 23240082 10.7554/eLife.00067 PMC3510455

[b40] Gibb B. & Greene E. C. Sliding to the rescue of damaged DNA. Elife 1, e00347 (2012). 23240090 10.7554/eLife.00347 PMC3510475

[b41] Iarovaia O. V. . Dynamics of double strand breaks and chromosomal translocations. Mol. Cancer 13, 249 (2014). 25404525 10.1186/1476-4598-13-249 PMC4289179

[b42] Mine-Hattab J. & Rothstein R. Increased chromosome mobility facilitates homology search during recombination. Nat. Cell Biol. 14, 510–517 (2012). 22484485 10.1038/ncb2472

[b43] Krawczyk P. M. . Chromatin mobility is increased at sites of DNA double-strand breaks. J. Cell Sci. 125, 2127–2133 (2012). 22328517 10.1242/jcs.089847

[b44] Mazina O. M. & Mazin A. V. Human Rad54 protein stimulates DNA strand exchange activity of hRad51 protein in the presence of Ca2+. J. Biol. Chem. 279, 52042–52051 (2004). 15466868 10.1074/jbc.M410244200

[b45] Chi P. . Functional interactions of meiotic recombination factors Rdh54 and Dmc1. DNA Repair 8, 279–284 (2009). 19028606 10.1016/j.dnarep.2008.10.012 PMC2650715

[b46] Chi P. . Yeast recombination factor Rdh54 functionally interacts with the Rad51 recombinase and catalyzes Rad51 removal from DNA. J. Biol. Chem. 281, 26268–26279 (2006). 16831867 10.1074/jbc.M602983200

[b47] Kwon Y. . ATP-dependent chromatin remodeling by the *Saccharomyces cerevisiae* homologous recombination factor Rdh54. J. Biol. Chem. 283, 10445–10452 (2008). 18292093 10.1074/jbc.M800082200 PMC2447626

[b48] Petukhova G., Sung P. & Klein H. Promotion of Rad51-dependent D-loop formation by yeast recombination factor Rdh54/Tid1. Genes Dev. 14, 2206–2215 (2000). 10970884 10.1101/gad.826100 PMC316899

[b49] Prasad T. K. . A DNA-translocating Snf2 molecular motor: *Saccharomyces cerevisiae* Rdh54 displays processive translocation and extrudes DNA loops. J. Mol. Biol. 369, 940–953 (2007). 17467735 10.1016/j.jmb.2007.04.005 PMC2705995

[b50] Santa Maria S. R., Kwon Y., Sung P. & Klein H. L. Characterization of the interaction between the *Saccharomyces cerevisiae* Rad51 recombinase and the DNA translocase Rdh54. J. Biol. Chem. 288, 21999–22005 (2013). 23798704 10.1074/jbc.M113.480475 PMC3724653

[b51] Wright W. D. & Heyer W. D. Rad54 functions as a heteroduplex DNA pump modulated by its DNA substrates and Rad51 during D loop formation. Mol. Cell 53, 420–432 (2014). 24486020 10.1016/j.molcel.2013.12.027 PMC4059524

[b52] Pezza R. J., Camerini-Otero R. D. & Bianco P. R. Hop2-Mnd1 condenses DNA to stimulate the synapsis phase of DNA strand exchange. Biophys. J. 99, 3763–3772 (2010). 21112301 10.1016/j.bpj.2010.10.028 PMC2998617

[b53] Seifert F. U., Lammens K., Stoehr G., Kessler B. & Hopfner K. P. Structural mechanism of ATP-dependent DNA binding and DNA end bridging by eukaryotic Rad50. EMBO J. 35, 759–772 (2016). 26896444 10.15252/embj.201592934 PMC4818761

[b54] Kim J. I. . RecA Protein from the extremely radioresistant bacterium *Deinococcus radiodurans*: expression, purification, and characterization. J. Bacteriol. 184, 1649–1660 (2002). 11872716 10.1128/JB.184.6.1649-1660.2002 PMC134872

[b55] Lusetti S. L. . C-terminal deletions of the *Escherichia coli* RecA protein. Characterization of *in vivo* and *in vitro* effects. J. Biol. Chem. 278, 16372–16380 (2003). 12598539 10.1074/jbc.M212917200

[b56] Lohman T. M., Green J. M. & Beyer R. S. Large-scale overproduction and rapid purification of the *Escherichia coli* ssb gene product. Expression of the ssb gene under lambda PL control. Biochemistry 25, 21–25 (1986). 3006753 10.1021/bi00349a004

[b57] Eggington J. M., Haruta N., Wood E. A. & Cox M. M. The single-stranded DNA-binding protein of *Deinococcus radiodurans*. BMC Microbiol. 4, 2 (2004). 14718065 10.1186/1471-2180-4-2 PMC331404

[b58] Pugh B. F., Schutte B. C. & Cox M. M. Extent of duplex DNA underwinding induced by RecA protein binding in the presence of ATP. J. Mol. Biol. 205, 487–492 (1989). 2538631 10.1016/0022-2836(89)90219-2

[b59] Robu M. E., Inman R. B. & Cox M. M. Situational repair of replication forks: roles of RecG and RecA proteins. J. Biol. Chem. 279, 10973–10981 (2004). 14701860 10.1074/jbc.M312184200

[b60] Garger S. J., Griffith O. M. & Grill L. K. Rapid purification of plasmid DNA by a single centrifugation in a two-step cesium chloride-ethidium bromide gradient. Biochem. Biophys. Res. Commun. 117, 835–842 (1983). 6199024 10.1016/0006-291x(83)91672-8

