# Supplementary material for: The cohesin-like RecN protein stimulates RecA-mediated recombinational repair of DNA double-strand breaks
Source: Nat Commun. 2017 May 17;8:15282. doi: 10.1038/ncomms15282 (PMC5442325; doi:10.1038/ncomms15282)
Supplement: Supplementary Information — Supplementary figures and supplementary references. [file ncomms15282-s1.pdf]

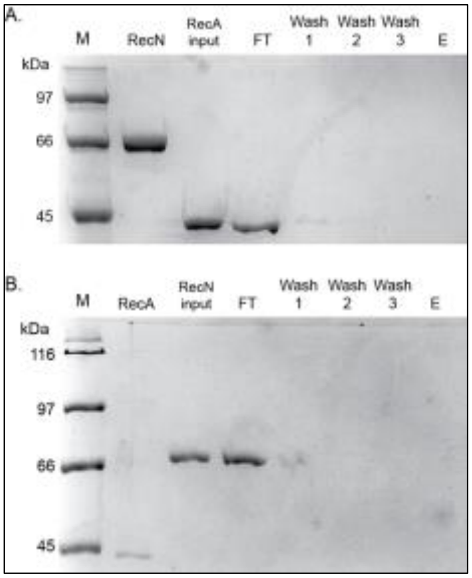

Supplementary Figure 1 - RecA and RecN protein pulldown control. Purified *D. radiodurans* RecA protein was added to a *D. radiodurans* RecN antibody-coupled resin (**panel A**) and purified *D. radiodurans* RecN protein was added to *D. radiodurans* RecA antibody-coupled resin (**panel B**) as described in the supplemental methods below. In each case the purified protein did not bind the resin and was collected in the flow-through (FT) fraction and a small amount in the first wash (Wash 1). No further protein was collected in subsequent wash steps (Wash 2 and Wash 3) or the elution step (E). These control experiments confirm that the pulldown assays showing that RecA and RecN co-eluting from the antibody-coupled resins in figure 3C are not due to non-specific interactions.

Pulldown experiments were carried out in the absence of linear duplex DNA as described in methods for figure 3C. Briefly, ~50 μg RecN antibody (Fig. S1A) or ~50 μg RecA antibody (Fig. S1B) were coupled to 100 μL of coupling resin. Purified RecA or RecN protein (to 2 μM) was incubated for 10 min at 37°C in Buffer N with 2.5 mM ATP in 80 μL final volume. The samples were diluted 1:3 in 1X Dulbecco's modified PBS buffer. Subsequent steps follow resin manufacturer instructions. The RecA diluted sample was added to the RecN antibody-coupled resin (Fig. S1A) or the RecN diluted sample was added to the RecA antibody-coupled resin (Fig. S1B) and incubated for 2 hours at 4°C. Unbound protein was collected as flow-through fraction and the resin was washed three times with 200 μL wash buffer and 200 μL elution buffer added as in methods for co-elution of RecA and RecN proteins. A 3K microcentricon (Millipore) was used to concentrate all samples (Flow-through, Wash 1, Wash 2, Wash 3, and Elution) to 20 μL. Protein samples were separated by 10% SDS-PAGE. The inverted gel images were obtained using a digital CCD camera with Foto/Analyst Pc Image software version 10.21 (Fotodyne).

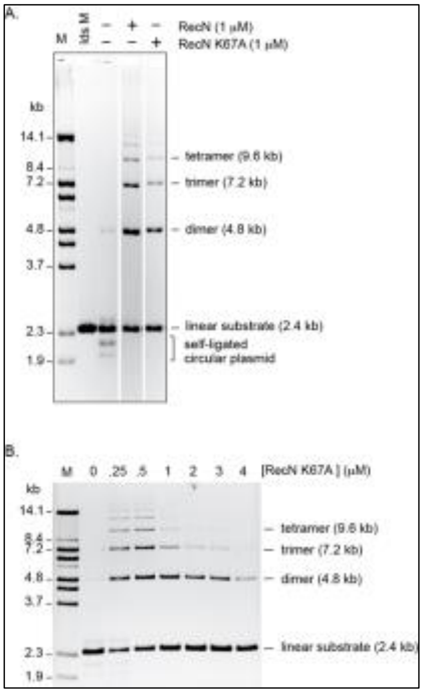

Supplementary Figure 2 - RecN K67A-dependent tethering of linear duplex DNA molecules. The RecN and RecN K67A proteins stimulate the ligase-dependent intermolecular ligation of linear duplex DNA. Ligation reactions were carried out in dilute conditions (as described in the methods below) such that the primary product of ligation in the absence of added RecN protein is self-ligated circular plasmid with various topoisomers of the intramolecular, covalently closed plasmid product. The wild-type RecN (1 μM, **panel A**) or RecN K67A mutant protein (1 μM, panel A or the indicated concentration, **panel B**) was incubated with 2.4 μM nt linear, 2.4 kb plasmid DNA substrate (marker, lds M) before the addition of *D. radiodurans* DNA ligase (to 4 nM). The ligated DNA products were purified from the dilute reaction mixture and analyzed in a 0.7% agarose gel. The migration of linear multimeric products relative to the BstE II digested lambda DNA marker standard (M) was used to identify the intermolecular ligation products as dimers (4.8 kb), trimers (7.2 kb), etc.

The addition of RecN or RecN K67A mutant stimulates the intermolecular ligation of linear duplex DNA (2.4 kbp) to form dimers, trimers, etc. As has been noted for the wild-type RecN protein [1], increasing concentrations of the RecN K67A protein inhibits the generation of large, higher-ordered, multimers (panel B). This is likely due to protein aggregation. However, DNA bridging is occurring at the concentration used in the D-loop formation assay of figure 2B.

DNA ligation assays were carried out as previously described [1]. Briefly, ligation reactions were carried out in a final reaction volume of 100 μL in buffer N and an ATP regeneration system (10 units mL^{-1} pyruvate kinase and 2.5 mM phosphoenolpyruvate). Wild-type RecN or RecN K67A mutant protein (final concentrations on figure) were incubated with 2.4 μM nt linearized pEAW3 DNA for 30 min at 37°C. After incubation, each reaction was provided with 12 μL of 10X DNA ligase reaction buffer (300 mM Tris-Cl (pH 8), 40 mM $MgCl_2$, 1 mM DTT, 260 μM NAD^+, 500 μg mL^{-1} BSA) and 4 nM *D. radiodurans* Ligase A and incubated at 30°C for 30 min. Ligation reactions were stopped by addition of 80 μL of termination buffer (20 mM Tris-HCl (80% +), 20 mM EDTA, 0.5% SDS) and deproteinized with 100 μg predigested proteinase K followed by 20 min incubation at 37°C. DNA from each reaction was recovered by phenol:chloroform:iso-amyl-alcohol solution (25:24:1) and precipitated with ethanol. The precipitated DNA was resuspended in 10 μL TE plus 5 μL of a solution containing 60 mM EDTA, 6% SDS, 25% (weight per volume) glycerol, and 0.2% bromphenol blue and electrophoresed in 0.7% agarose gels in 0.5X TBE buffer. DNA was visualized by ethidium bromide staining and inverted images were obtained using a Fotodyne image system equipped with a digital CCD camera and Foto/Analyst PC image software v10.21.

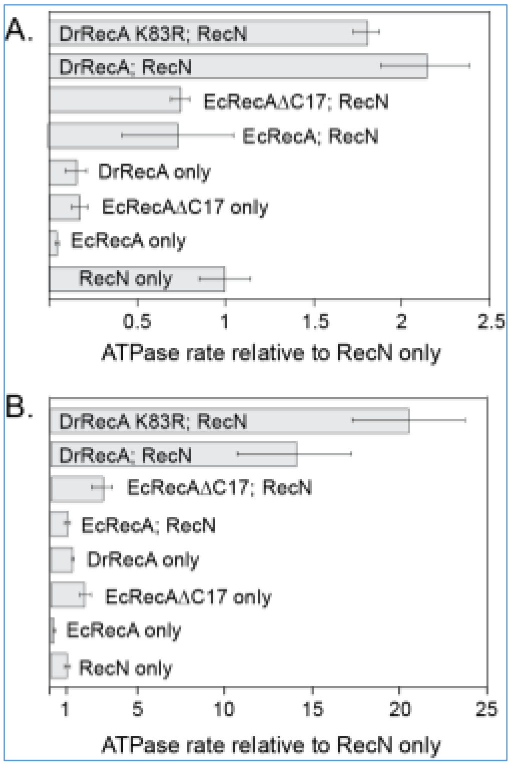

Supplementary Figure 3 - The RecN ATPase activity is stimulated by *D. radiodurans* (Dr) RecA but not *E. coli* (Ec) RecA protein. Using the same conditions of figure 4, reaction 5 (**panel A**), we compared the RecN ATP hydrolysis rate in the absence or presence of a low concentration (0.5 μM) of RecA proteins: wild-type DrRecA, DrRecA K83R mutant protein, wild-type EcRecA, or EcRecAΔC17 mutant protein. The RecA proteins, where indicated, were incubated with linearized pEAW324 plasmid (50 μM nt) before the addition of 2 μM RecN protein. The steady-state rates were measured and graphed relative to the rate of RecN ATP hydrolysis in the absence of added RecA protein (RecN only). The error bars represent the standard deviation of the relative rate (the standard deviation of the average rate divided by the average rate) of six or seven independent experiments. The experimental procedure was the same as for figure 4, reaction 5 except for the concentration of RecA used. The RecAΔC17 was purified as described [2].

A low concentration (0.5 μM) RecA protein was used so that the rate of ATP hydrolysis measured in the absence of RecN was minimized. When the DrRecA or DrRecA K83R mutant protein is included, the RecN ATP hydrolysis rate increases ~2-fold under these lower (than in the experiments of Fig. 4) RecA concentration conditions. EcRecA protein nucleates slowly onto duplex DNA. Therefore, we also included an experiment with the EcRecAΔC17 mutant protein that has been shown to nucleate rapidly onto duplex DNA [2]. When the EcRecA or EcRecAΔC17 mutant protein is included, the measured rate of RecN ATP hydrolysis is similar to that of RecN alone.

The experiments of panel A and figure 4 are carried out under the conditions previously optimized for RecN activity [1]. However, the DNA strand exchange and D-loop formation assays of figure 1 and 2 are carried out under different solution conditions. The major difference in conditions (see methods for figure 4) is that the RecN-optimized solution (Buffer N) contains 1% polyethylene glycol (PEG) and 17.5 mM magnesium acetate (panel A and Fig. 4) and the RecA-optimized solution (Buffer A) contains 10 mM magnesium acetate and no PEG. We therefore compared the RecN ATP hydrolysis rate in the presence and absence of 0.5 μM RecA proteins under the RecA-optimized solution conditions (**panel B**). Under the RecA-optimized conditions we observe a very low rate (~ 2 μM min^{-1}) of RecN ATP hydrolysis in the absence of a RecA protein. When the DrRecA or the DrRecA K83R proteins are included, we observe a 15 to 20-fold stimulation of the rate of RecN ATP hydrolysis. This is consistent with the stimulation observed in figure 5. However, we observe an only additive effect when the EcRecA or EcRecAΔC17 mutant protein is included suggesting the *E. coli* proteins do not stimulate RecN ATP hydrolysis.

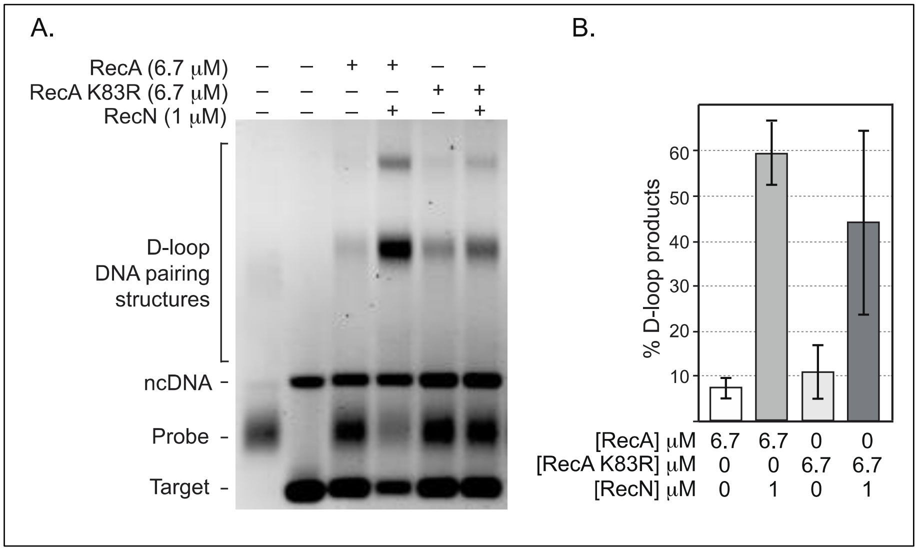

Supplementary Figure 4 - RecA K83R mutant protein catalyzes D-loop formation. RecA filaments formed on the linear duplex plasmid DNA substrate containing 150 nt 3' ssDNA overhangs (Probe) promote strand invasion within the 2.4 kb, homologous, supercoiled plasmid DNA (Target). RecA exchanges the homologous strands forming D-loop structures. These descriptions, Probe, Target, and D-loop, reflect the agarose gel labels used here and are shown schematically in figure 2A. RecA or RecA K83R mutant (6.7 μM) was incubated in Buffer A plus an ATP regeneration system (see Methods) with 20 μM Probe DNA for 10 min at 37°C. ATP (3 mM) and 1 μM RecN, as indicated at the top of each lane, were incubated for an additional 10-min prior to starting the reaction with the addition of 20 μM homologous target DNA. All reactions were incubated for 45 min. Reactions were stopped by adding 2x Loading Stop Buffer and proteinase K (to 1.25 mg mL^{-1}). Samples were subjected to electrophoresis in 0.8% agarose gels with TBE buffer, stained with ethidium bromide, and exposed to UV light. The inverted gel images were obtained using a digital CCD camera with Foto/Analyst PC Image software version 10.21 (Fotodyne). **B)** Quantification of amount of D-loop pairing structures formed by 6.7 μM RecA or RecA K83R mutant protein in 45 min in the presence or absence of 1 μM RecN protein. The D-loop products are defined as the sum of all DNA band intensities in a particular lane that correspond to the mobility of the D-loop DNA pairing structures identified in panel A that were detected by the TotalLab gel quantification software. This sum was divided by the sum of all band intensities (except the band corresponding the ncDNA) in the same lane. Error bars represent the standard deviation of 3 independent experiments.

Supplementary References:

1. Reyes, E.D., et. al. RecN is a cohesin-like protein that stimulates intermolecular DNA interactions in vitro. *J Biol Chem* 285, 16521-9 (2010).
2. Lusetti, S.L., et. al. C-terminal deletions of the *Escherichia coli* RecA protein: Characterization of in vivo and in vitro effects. *J Biol Chem* 278, 16372-80 (2003).
